# Nanoplasmonic pillars engineered for single exosome detection

**DOI:** 10.1371/journal.pone.0202773

**Published:** 2018-08-24

**Authors:** Deepa Raghu, Joseph A. Christodoulides, Marc Christophersen, Jinny L. Liu, George P. Anderson, Michael Robitaille, Jeff M. Byers, Marc P. Raphael

**Affiliations:** 1 Materials Science and Technology Division, Naval Research Laboratory, Washington, D.C., United States of America; 2 Space Sciences Division, Naval Research Laboratory, Washington, D.C., United States of America; 3 Center for Biomolecular Science & Engineering, Naval Research Laboratory, Washington, D.C., United States of America; University of Massachusetts Boston, UNITED STATES

## Abstract

Exosomes are secreted nanovesicles which incorporate proteins and nucleic acids, thereby enabling multifunctional pathways for intercellular communication. There is an increasing appreciation of the critical role they play in fundamental processes such as development, wound healing and disease progression, yet because of their heterogeneous molecular content and low concentrations *in vivo*, their detection and characterization remains a challenge. In this work we combine nano- and microfabrication techniques for the creation of nanosensing arrays tailored toward single exosome detection. Elliptically–shaped nanoplasmonic sensors are fabricated to accommodate at most one exosome and individually imaged in real time, enabling the label-free recording of digital responses in a highly multiplexed geometry. This approach results in a three orders of magnitude sensitivity improvement over previously reported real-time, multiplexed platforms. Each nanosensor is elevated atop a quartz nanopillar, minimizing unwanted nonspecific substrate binding contributions. The approach is validated with the detection of exosomes secreted by MCF7 breast adenocarcinoma cells. We demonstrate the increasingly digital and stochastic nature of the response as the number of subsampled nanosensors is reduced from four hundred to one.

## 1. Introduction

Exosomes are cell-derived nanovesicles that mediate intercellular communication by transporting biomolecules such as lipids, proteins, and RNA from one cell to another, thereby regulating post-transcriptional modification in the recipient cell [1–6]. Exosomes are of great significance due to their multifunctional attributes and potential for early diagnosis and treatments of diseases such as cancer and metabolic disorder [7–9]. For instance, some cancer cells secrete higher amounts of exosomes compared to healthy cells [10], and studies have shown that exosomes can be used for the detection of cancer tumors such as breast, prostate, ovarian, and hematologic malignancies [11–15]. Recently, it has been realized that exosomes have clinical potential as sensitive, non-invasive biomarkers due to their presence in circulating body fluids such as saliva and blood, as well as having applications in targeted drug delivery [16]. Despite this growing research interests, the accepted methodologies for isolating exosomes from extracellular components such as proteins, protein aggregates, ectosomes and lipids are still continuing to evolve [17–19]. This is due to the heterogeneity of their molecular content and protein expression, their small size (~50–200 nm), and their fairly large size distribution that overlaps with other classes of membranous extracellular vesicles (EVs), such as ectosomes and apoptic bodies [20], making perceived exosome detection difficult to discern from other potential supernatant components.

A driving goal of exosome research is the ability to perform “liquid biopsies” for early detection of a host of diseases known to be associated with exosomes. The field is striving to increase both detection sensitivity and throughput, with the ultimate goal being a rapid, highly multiplexed assay with single exosome sensitivity. Standard techniques optimized for protein detection and characterization, such as ELISA and surface plasmon resonance (SPR), are often not well-matched to the size, complexity and low concentrations required for exosome work. As a result, a number of groups have been developing sensors tailored specifically towards exosome detection. Single Particle Interferometric Reflectance Imaging Sensors (SP-IRIS) were recently shown as a label-free method to detect populations of exosomes in a multiplexed format but is not a real-time technique [21]. Single exosome detection has been recently achieved via frequency-locked microtoriod optical resonators [22] and optical trapping [23], however these experimental platforms are not readily multiplexed making the analysis of large sample populations cumbersome. Localized surface plasmon resonance (LSPR) is an ideal platform for multiplexed exosome detection because of its real-time nature and the ability to tailor sensor sizes to match that of individual exosomes for optimal single-exosome sensitivity. It has been shown that fabricated LSPR nanoholes yield a signal to noise ratio similar to that of conventional SPR [24]. A number of recent studies have shown the applicability of both LSPR and SPR to the detection of exosomes [25, 26]. One recent approach in particular employed 200 nm diameter plasmonic nanoholes for exosome detection (nPLEX), drastically improving the lower-limit detection down to approximately 3000 exosomes [27].

Here we present a localized surface plasmon resonance imaging (LSPRi) platform which improves the limit of detection by three orders of magnitude—down to the single exosome limit. The approach utilizes lithographically patterned gold nanosensors that have multiple design features geared to improve exosome detection confidence and limits. First, the gold nanosensors are size-matched to the approximate diameter of a single exosome, which allows distinction between detecting smaller proteins/molecules and larger EVs via the LSPRi temporal signature. Second, the gold sensing elements are elevated atop quartz nanopillars, which reduces background contributions from non-specific binding of the underlying substrate. Finally, the use of nanoplasmonic sensing, a modality with sensitivity that only extends tens of nanometers into the solution, gives increased confidence in the detection of high affinity sensor-surface binding events. The chip, fabricated using a collection of nano- and microfabrication techniques, employs thousands of individual nanosensors, enabling highly multiplexed data collection and readily integrates onto a standard optical microscope. The design was validated by biofunctionalizing the nanosensors with anti-CD63 antibodies targeted towards the tetraspanin CD63 membrane bound proteins present in exosomes secreted by MCF7 breast adenocarcinoma cells. By subsampling the imagery to monitor sequentially fewer nanopillars, down to the individual nanopillar limit, the data revealed increasingly digital responses occurring stochastically in time and space as expected for single exosome binding in the sub-femtomolar concentrations of exosomes used in these studies.

## 2. Experimental methods

### 2.1 Electron beam lithography of gold nanopillars

**Fig 1:**
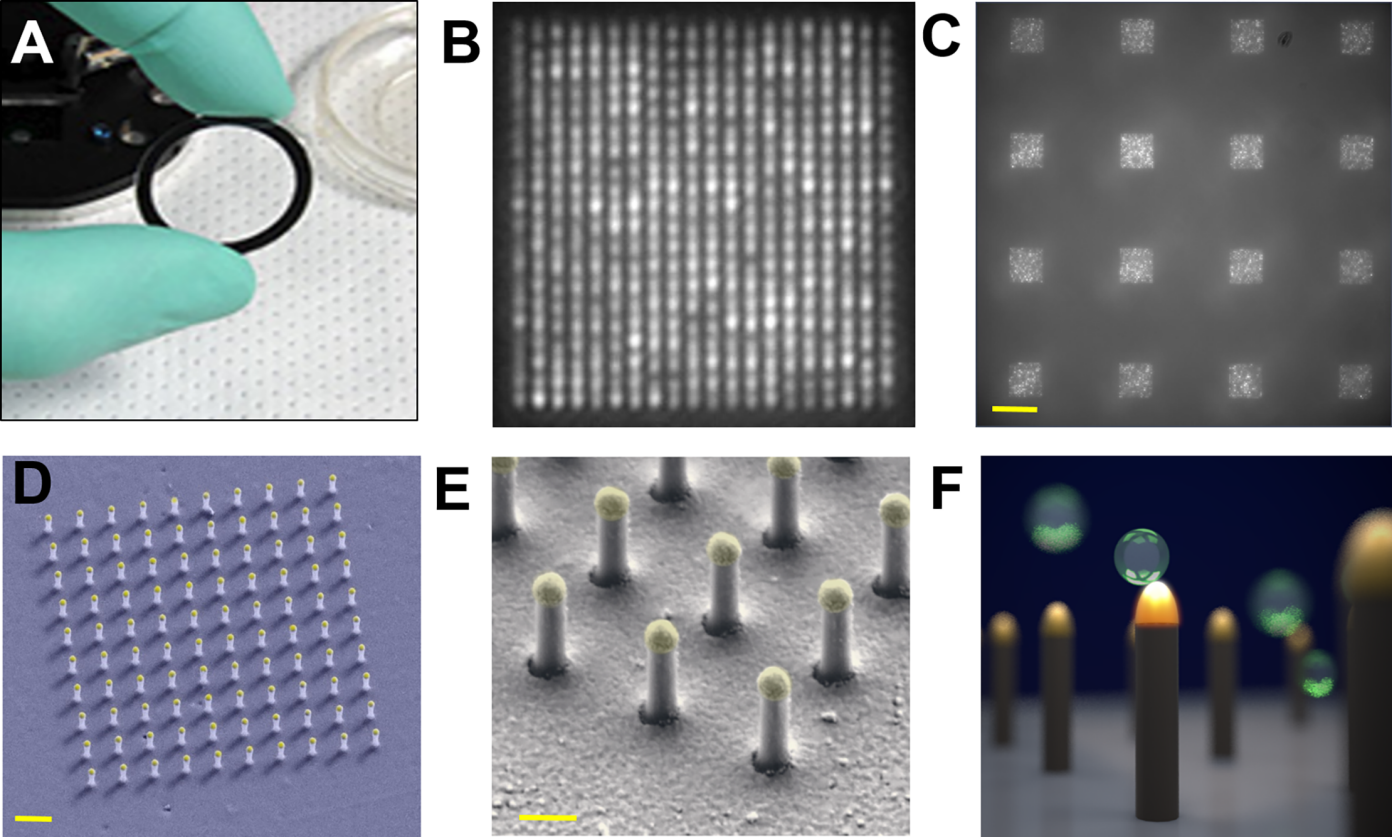
(A) 25.4 mm diameter LSPRi sensor chip. (B) LSPRi image of a 20 × 20 array, with a pitch size of 600 nm scale bar: 1 μm. (C) LSPRi image of sixteen arrays in the FOV taken using 100X / 1.4 NA objective, each consisting of 400 plasmonic nanopillars in a 20 × 20 square lattice and 500 nm pitch, scale bar: 10 μm. (D) False colored SEM image of a 10 × 10 nanopillar array, scale bar: 1 μm. (E) High-magnification false colored SEM image showing detailed view of individual nanopillars, scale bar: 200 nm. (F) Diagram illustrating size matching of individual nanopillars diameter (d = 90 nm) to that of exosomes (~50 nm < d < 200 nm), allowing digitized exosome detection while also elevating the sensor to minimize background contributions from the substrate.

Number 1.5, 25.4 mm diameter quartz coverslips (SPI Supplies) were used for patterning the nanopillars (**Fig 1A**). The coverslips were cleaned with piranha acid and spin coated with a bilayer resist as previously described [28]. The resist was coated with a 25 nm thick aluminum (Al) layer for charge dissipation via electron-beam evaporation (Temescal). After electron beam exposure (Raith 150) the top Al layer was removed by a two minute aqueous 5% tetramethyl-ammonium hydroxide (TMAH) etch. The resist was developed for 60 sec in a 1:2 isopropanol (IPA) to methyl isobutyl ketone (MIBK) solution. After developing, the chip was rinsed in IPA for 30 sec followed by a “descum” oxygen plasma cleaning step for 20 sec at 30 mTorr.

The gold for nanoplasmonic sensing was deposited by electron beam evaporation using a Temescal system to a thickness of 130 nm. The resist was lifted off by soaking the sample for 24 hrs in acetone followed by acetone and IPA rinses. The patterned quartz coverslip was then dry etched in a reactive ion etcher (RIE) from Oxford Instruments (PlasmaPro 100) with a gas mixture of O_2_ and CHF_3_ at flow rates of 30 standard cubic centimeters per minute (sccm) for O_2_ and 50 sccm for CHF_3_. The pressure was kept at 40 mTorr, and the forward power set at 100 W, resulting in a voltage to ionize the gas mixture of 375–380 V. The RIE process lasted 15–25 min with a quartz etching rate of 35 nm/min and a Au etch rate of 2 nm/min. After etching, the quartz chip was removed from the silicon carrier wafer and rinsed with acetone and IPA. After RIE etching the remaining gold thickness was 75–85 nm as estimated from the calibrated etch rate and verified with scanning electron microscope (SEM) imagery.

We have made LSPRi sensor chips with a diameter of 25.4 mm (**Fig 1A**). Each sensor chip surface was fabricated with nanopillar arrays, and each nanopillar array consisted of either 10 × 10 or 20 × 20 evenly spaced nanopillars with pitches of either 500 nm or 600 nm (**Fig 1B**). The arrays were patterned in a square matrix geometry and separated by 25 μm edge-to-edge (**Fig 1C**). **Fig 1D** shows a false-colored, tilted SEM micrograph of a 10 × 10 nanopillar array, while **Fig 1E** shows a close up of individual nanopillars. A single nanopillar base had a typical diameter of 90 nm and a total height of 490 nm including the approximately 80 nm gold cap, engineered for single exosome detection (**Fig 1F**).

### 2.2 Optical setup

The optical and microfluidic set up for LSPRi has been previously described [28–30]. Briefly, the chip was loaded into a microfluidic assembly and placed on an inverted Zeiss Axio Observer microscope. The exosome solution was introduced over the functionalized chip using a peristaltic pump (Instech P720). The imagery data was collected using a 100X / 1.4 NA objective and thermoelectrically cooled 16 bit CMOS camera (FLASH 4.0, Hamamatsu) operated in 1 ×1 binning mode. The imagery data was recorded every 20 sec with an exposure time of 1.3 sec for 2 hrs. The field of view incorporated sixteen nanoarrays each consisting of 400 nanopillars (20 × 20), giving a total of 6400 individually addressable nanosensors per image (**Fig 1C**). With this optical configuration each nanopillar was in registry with 9 × 9 pixels of the CMOS camera (6.5 μm/pixel). Nanoplasmonic spectra was simultaneously collected by projecting the focused image of a single array on to an optical fiber and measured with CCD-based spectrophotometer (QE65000, Ocean Optics) with an integration time of 5 sec.

### 2.3 Exosome characterization

SPRi and LSPRi experiments were conducted on MCF7 secreted exosomes purchased from Systems Biosciences, Inc. (EXOP-100A-1, SBI). The company harvests exosomes from cell lines grown in exosome-depleted FBS (Exo-FBS, SBI) and purified using their ExoQuick-TC product. To remove any agglomerates that occurred during shipping, we conducted an additional centrifugation on the as-received vials at 1500 x g for 5 sec. The PBS buffer (Sigma) used for dilutions was two-stage filtered through 0.22 μm (CELLTREAT) and 0.02 μm filters (Whatman, Anotop), respectively.

**Fig 2:**
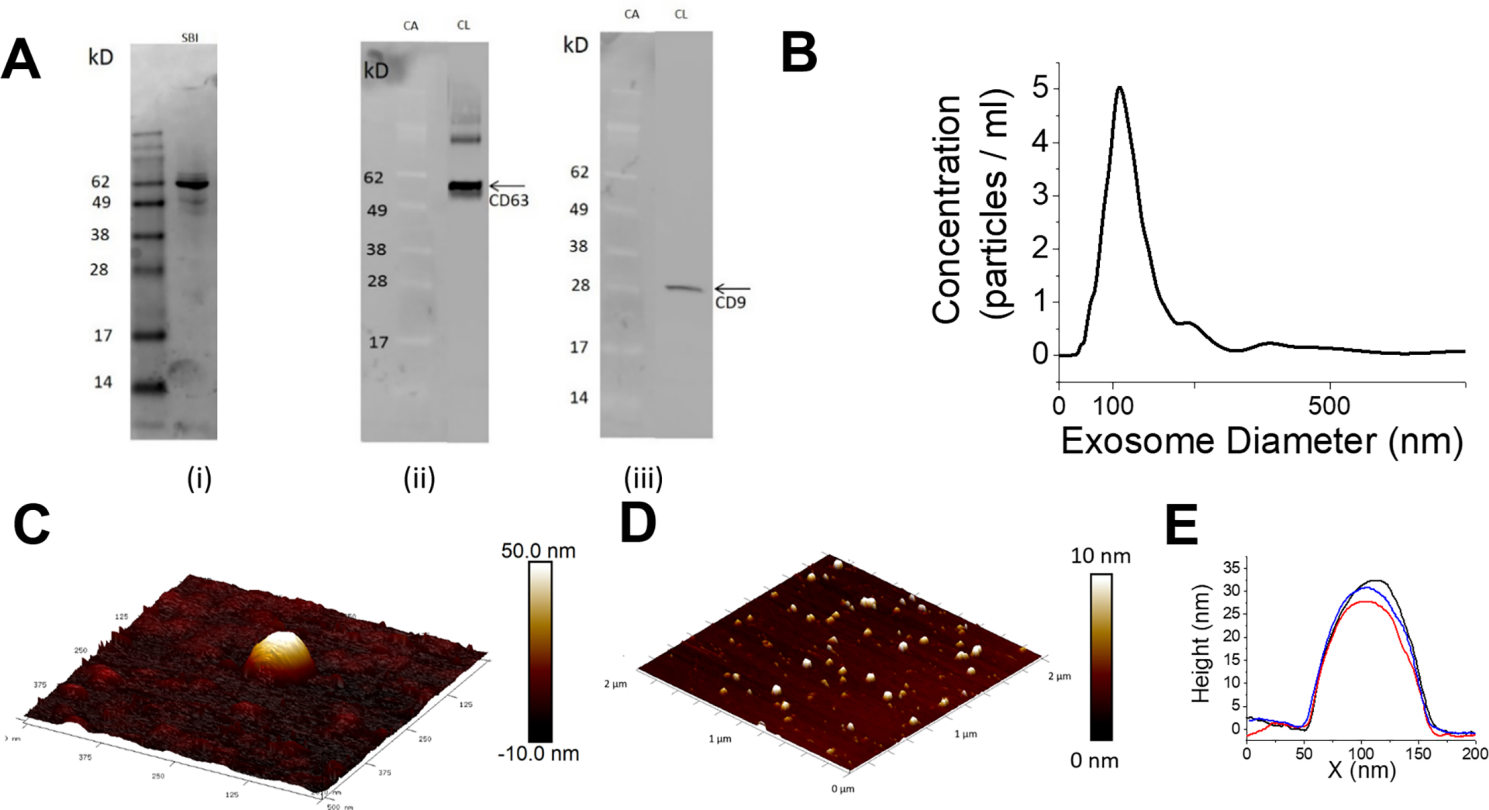
MCF7 exosome characterization. (A) Western blot analysis of exosomes (i) MCF7 exosome protein profile (ii) Colorimetric image (CA) lane displays standard weight markers and chemiluminescent image (CL) lane shows CD63 detection near 55 kD. (iii) Colorimetric image (CA) lane displays standard weight markers and chemiluminescent image (CL) lane shows CD9 detection near 28 kD. (B) Nanosight analysis of MCF7 exosomes showing the size distribution. (C) AFM image of a single MCF7 exosomes in 0.1 M PBS on freshly cleaved mica. (D) AFM image of larger populations of MCF7 exosomes. (E) Representative cross-sections of several exosomes taken from AFM scans.

The exosomes’ protein profile was characterized using western blot with particular focus on the presence of CD63 and CD9. Approximately 3 × 10^7^ exosomes were loaded onto 4–12% NUPAGE gel and separated at 135 V for 1 hr 35 min in the presence of MES SDS buffer (Thermo Fisher Scientific Inc.). The proteins in the gel were subsequently transferred to Polyvinylidene difluoride (PVDF) (Thermo Fisher Scientific Inc.) running at 60 V for 2 hrs at 4°C in Tris-glycine methanol buffer (Tris-HCL: 19.2 mM; Glycine: 192 mM; methanol: 20%). At the end of the transfer, blots were briefly washed with 1X TBST buffer (10 mM Tris pH7.6, 15.4 mM NaCl, 0.1% Tween 20), followed by incubation with 5% milk in TBST buffer (TBSTM) at room temperature for 1 hr. Blots were further incubated at room temperature for another hour in TBSTM containing anti-human CD63 antibody (SBI) at 1:1000 dilution. Blots were rinsed 3 times for 15 min with TBSTM, followed by the incubation in HRP conjugated secondary antibody (SBI). Subsequently blots were rinsed 3 times for 5 min with TBST and the chemiluminescent reagent (Thermo Fisher Scientific Inc.) was then applied to blots. The images were captured using a CCD-based Bio-Rad XRS+ system. **Fig 2A** (i) shows the exosome protein profiles with **Fig 2A** (ii-iii) highlighting the bands consistent with the weights and specificities for CD63 and CD9.

The exosome size distribution was characterized using a Nanosight NTA system (Malvern Instruments). For the Nanosight measurements, the exosomes were diluted to an approximate concentration of 1 × 10^9^ exosomes/ml in filtered 0.1 M PBS. **Fig 2B** shows a typical measurement which resulted in a mean diameter of 173 ± 130 nm and a mode of 112 nm.

Exosome size and morphology were characterized by Atomic Force Microscopy (AFM, Dimension Icon, Bruker). For all AFM imaging, the purified exosomes were diluted to approximately 1 × 10^9^ exosomes/ml, 50uL of which was drop coated on freshly cleaved mica (V1 grade, Ted Pella) for 5 min. Approximately 300 uL of filtered PBS (0.02 μm filter, Whatman) was then added to form a liquid cell for fluid imaging. Soft cantilever probes (MLCT-E & Scanasyst Air, Bruker) were utilized under Peakforce Tapping to allow for tight control of the tip-sample interaction forces (50–300 pN). Topographic height images were recorded at 512 × 512 pixels at a scan rate of 1 Hz. Under these conditions, the measured exosomes had a distribution of diameters of 50–150 nm with spherical/ellipsoidal morphology. **Fig 2C** shows a 500 nm x 500 nm topography scan of a single exosome immobilized on a mica surface under PBS buffer, Fig 2D shows a larger 2μm x 2μm scan of several exosomes under similar conditions, as well as representative cross sections from different exosomes in **Fig 2E**. A control scan is shown in **S3 Fig**.

## 3. Results and discussion

### 3.1 SPRi-based surface functionalization and kinetics characterization

Surface functionalization conditions for high specificity were optimized using a commercial SPRi instrument (Biorad ProteOn XPR36). The instrument is capable of multiplexing 6 ligand lanes and 6 analyte lanes, enabling the efficient characterization of a battery of potential antibody candidates for exosome detection. SPRi and LSPRi both work on the principle of plasmonic resonance imaging with a primary difference being the sensing area: 250,000 μm^2^ for a single SPRi sensor and 0.36 μm^2^ for one of our Au capped nanopillars. This allowed us to compare and contrast the signal and noise characteristics of the two instruments when applying the same biofunctionalization and measurement protocol as detailed below.

The SPRi surface functionalization procedure consisted of pre-cleaning the Bio-Rad chips using hydrogen plasma ashing at 40 W, 300 mTorr in a mixture of 5% hydrogen and 95% argon. The bare Au surface was functionalized by immersing the chip in a variable ratio of SH-(CH2)_8_-EG_3_-OH (SPO): HS-(CH_2_)_11_-EG_3_-COOH (SPC) (Prochimia Surfaces Sp.) for 18 hrs in order to form a self-assembled monolayer (SAM) as previously described [31]. The chip was rinsed with ethanol and dried under nitrogen gas. The SPO component was included to reduce non-specific binding, and after a range of two-component ratios were investigated, we found a SPO:SPC ratio of 100:1 exhibited minimal non-specific binding without significantly affecting sensitivity (**S2 Table**, **S2 Fig**).

Next, the surface was activated with 33 mM:133 mM ratio of N-hydroxysulfosuccinimide (sulfo-NHS) and 1-ethyl-3-[3-dimethylaminopropyl] carbodiimide hydrochloride (EDC) (Thermo Fisher Scientific) in the SPRi instrument at a flow rate of 30 μl/min for 300 sec. Antibodies were diluted in pH 6.0 phosphate buffer and introduced at a concentration of 5 μg/ml and flow rate of 30 μl/min for 300 sec. Unreacted SPC was deactivated with 0.1 M ethanolamine at a flow rate of 30 μl/min for 300 sec. Exosomes, diluted in PBS to a concentration of 15 μg/ml (nanodrop, Thermo) or approximately 10^5^ exosomes/ml, were introduced for the association phase at a flow rate of 25 μl/min for 982 sec followed by a dissociation phase consisting of only buffer for 1200 sec. The functionalization of LSPRi chips proceeded in a similar manner except that the solutions for ligand crosslinking were drop coated and manually rinsed, and in the final step, exosomes in PBS were introduced over the LSPRi chip using the peristaltic pump assembly at a flow rate of 250 μl/min.

A total of 20 antibodies were screened for maximizing the specific plasmonic response to MCF7 exosomes versus a control antibody functionalized surface (**S1 Table**). From these, the highest signal-to-background results were obtained from rabbit anti-human CD63 (SBI) and mouse anti-human CD9 (BD Biosciences) vs the control rabbit IgG antibody targeted to Staphylococcal enterotoxin B. The anti-CD63 had a higher response to the MCF7 exosomes (33%) than anti-CD9 and also exhibited high binding affinity with an equilibrium dissociation binding constant of 17 pM (**S1 Fig**) and was used for all LSPRi studies reported in this work.

### 3.2 LSPRi-based surface functionalization and characterization

**Fig 3:**
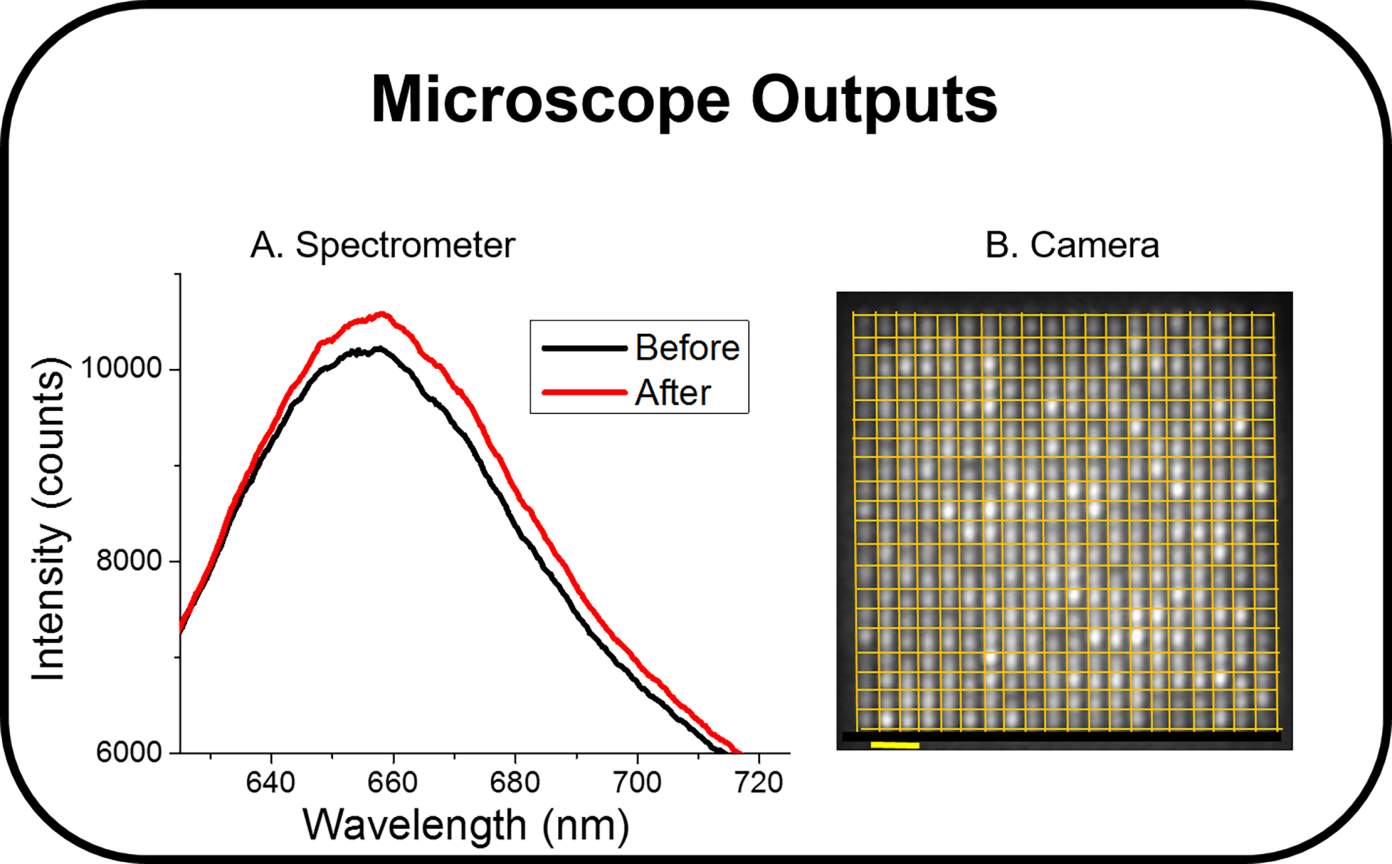
Spectral LSPR data versus LSPRi. (A) LSPR spectra from a 400 nanopillar array taken before (black) and after (red) introducing the exosomes (10^5^ exosomes/ml). The spectrometer’s spatial resolution and integration time were 200 μm^2^ and 5 sec, respectively (B) CMOS Image of the same array allowing for single nanopillar resolution, 0.5 μm^2^ resolution, and a faster integration time of 1.3 sec. Grid overlay denotes region of interest for individual nanopillar intensity measurements. In LSPRi the nanopillars brighten in response to analyte as a result of the shifting spectra. Scale bar = 1 um.

Based on the SPRi and western blot characterization studies, the anti-CD63 antibody was chosen for the nanoplasmonic detection of exosomes (**Fig 2A**, **S1 Fig**). Nanoplasmonic pillars were designed with a diameter of 90 nm, or roughly half the mean exosome diameter, to accommodate one or zero exosomes and increase the probability of a digital response (**Fig 1E**). In a typical LSPR application, the resonance peak is spectroscopically monitored for red-shifts as the analyte is introduced, indicating a change in the local index of refraction near the nanostructure’s surface due to binding events. Such a measurement is shown in **Fig 3A**, in which a solution of 10^5^ exosomes/ml induced a 2 nm peak red shift as well as an overall increase in the scattered peak intensity. The signal is the aggregate of reflected light from a single array of 400 nanopillars which was the limit of the spectrometry setup’s spatial resolution (200 μm^2^).

**Fig 4:**
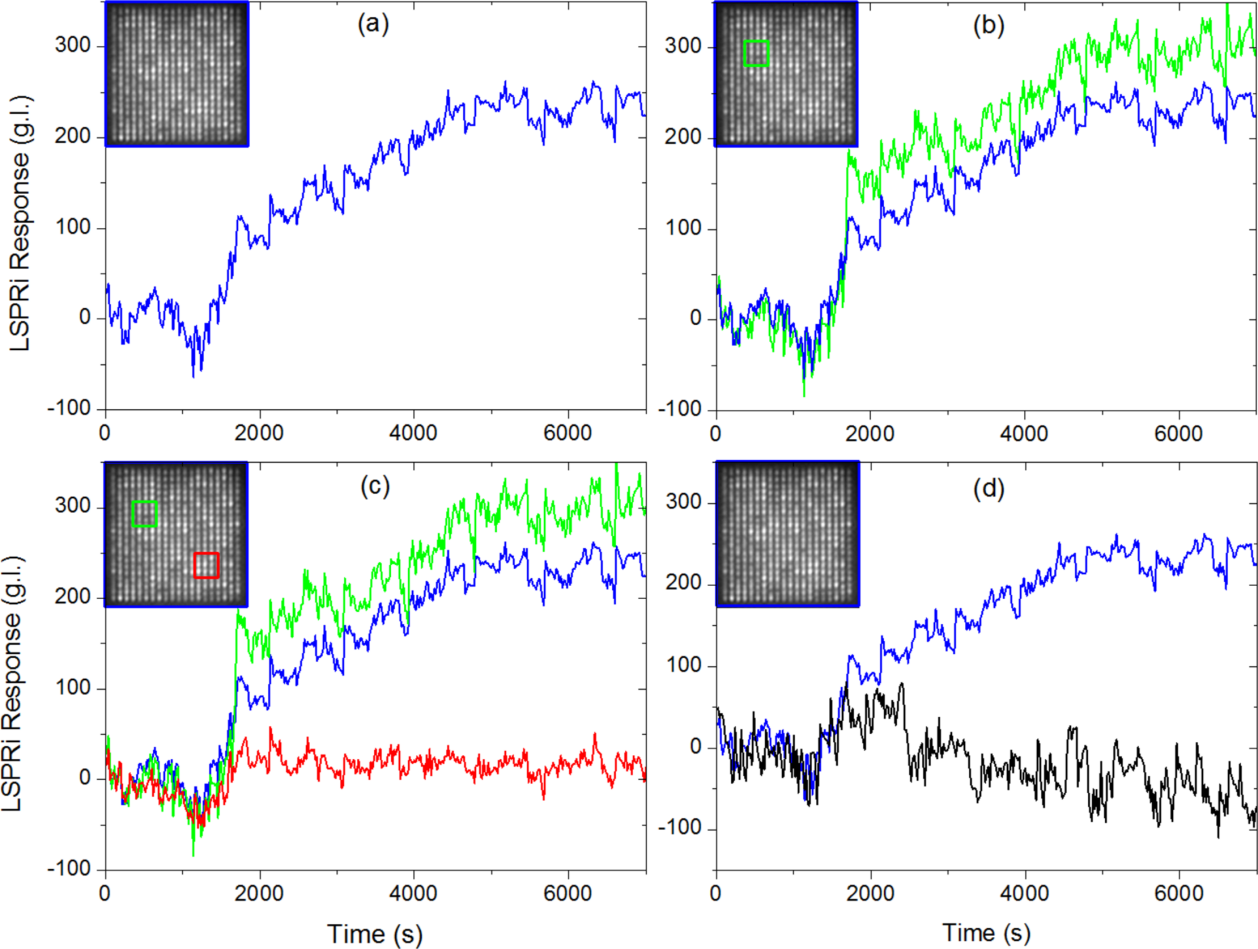
Subsampled LSPRi time series from a 400 nanopillar array (background subtracted). The blue, green and red squares on the inset images encompass the subsampled nanopillars, the average response of which is plotted in the corresponding color. For all panels, 1 × 10^5^ exosomes/ml was introduced at t ~ 1200 sec. (A) Spatially averaged time course of the entire 20 × 20 array (144 μm^2^) in blue. (B) Spatially averaged time course of the entire 20 × 20 array (144 μm^2^) and the 4 × 4 subarray (5.8 μm^2^) outlined in green (C) Spatially averaged time course of the entire 20 × 20 array (144 μm^2^), the 4 × 4 subarray (5.8 μm^2^) outlined in green and the 4 × 4 subarray (5.8 μm^2^) outlined in red. (D) Spatially averaged time course of the entire 20 × 20 array (144 μm^2^) and a separate control experiment (black data) on the same array functionalized with the IgG control antibodies.

In contrast, our LSPRi measurements were made by imaging the nanopillars on a CMOS camera as shown in **Fig 3B**. Exosome detection was manifested by increased nanostructure brightness resulting from the spectral shift [30]. The camera resolution and field of view incorporated 6,400 resolvable nanopillars (16 arrays with 400 nanopillars each), enabling highly multiplexed exosome detection with single nanopillar resolution (0.36 μm^2^). **Fig 4A** shows a typical LSPRi exosome binding response from one such anti-CD63 functionalized array (400 nanopillars, 144 μm^2^), illustrating the sensitivity of the LSPRi platform when a solution of 10^5^ exosomes/mL (sub-femtomolar concentration) was introduced into the fluidics chamber at t = 1200 sec. By subsampling the imagery, we are able to infer analyte size information as follows: biomolecular analytes significantly smaller than the nanosensors (*i*.*e*. proteins) diffuse quickly enough that all nanosensors in the field of view will exhibit the same temporal response [31]. For larger analytes, such as exosomes, a more stochastic spatial and time distribution of digital responses is expected due to the similar sizing of the exosomes and nanosensors.

**Fig 4B** highlights a subsampled array of 16 nanopillars (5.8 μm^2^) in the top left quadrant of the array (green square and data), which shows both a higher and sharper response than the co-plotted 400 nanopillar curve (blue square and data). In contrast, the 16 nanopillars in the lower right quadrant of the array in **Fig 4C** (red square and data) gave no resolvable response. These results are consistent with localized, discrete detection events of a low-concentration and larger-sized analyte, while the exosome specificity was confirmed by a separate control antibody experiment shown in **Fig 4D** (black data).

**Fig 5:**
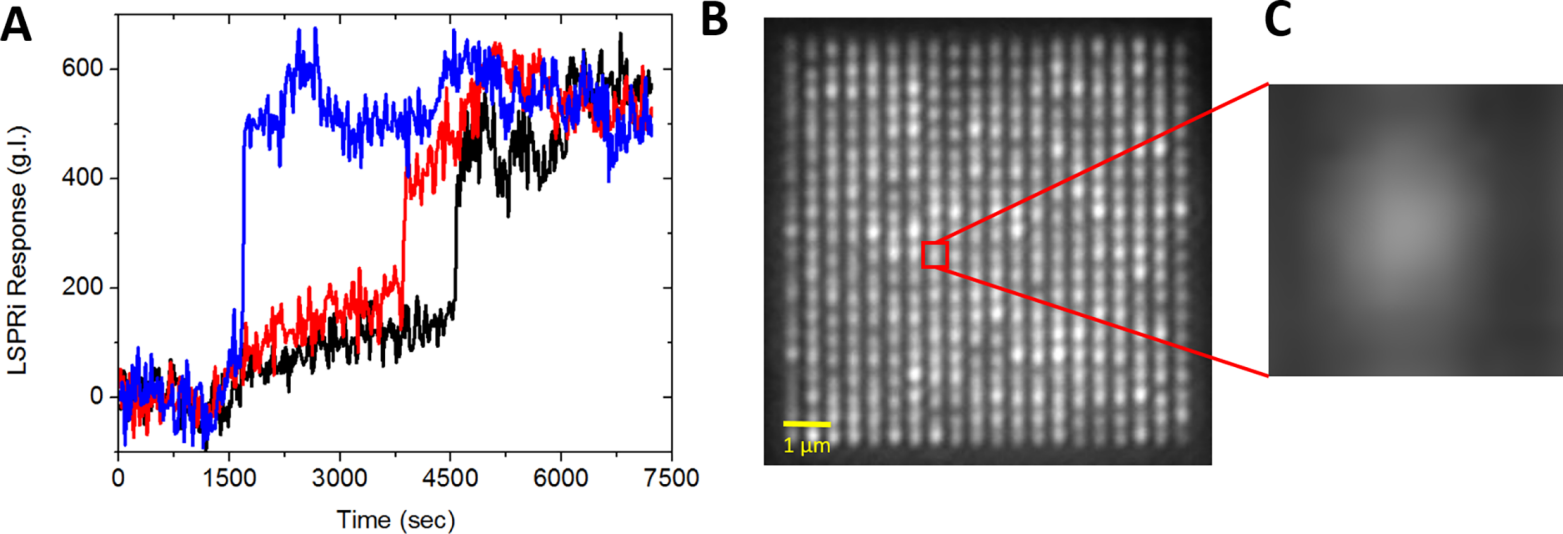
(A) Spatially averaged data from three individual nanopillars (0.36 μm^2^) in blue, red, and black, respectively (background subtracted). (B) LSPRi of 20 x 20 nanosensor array highlighting a subsampled area for individual nanostructure measurements. Scale bar 1 μm. (C) Zoomed in view of the single nanosensor measurement area. 1 × 10^5^ exosomes/ml were introduced at t ~ 1200 sec. The sharp jumps and stochastic nature of the detection events are consistent with single exosome detection.

The fact that sharp temporal responses are observed from some nanopillars while only a few microns away others give no discernable response is made possible by reducing the sensor size to approximately that of the exosome. We were not able to completely isolate the signal from individual nanopillars due to diffractive effects, which created bleed-through from neighboring nanopillars. However, we did observe temporal sharpening in the exosome-detection response when subsampling individual nanopillars, indicating that crosstalk from diffraction made only second-order contributions (**Fig 5A**). In this experiment, individual nanopillars were subsampled while injecting 10^5^ exosomes/ml over anti-CD63 functionalized nanopillars. The data shows three separate nanopillars in a single array which exhibited sharp responses at t = 1500 sec, 3700 sec, and 4200 sec, respectively. The digital nature of the response amongst individual nanopillars is consistent with single exosome detection, as is the stochastic nature of the detection in which the time between binding events spans tens of minutes. In all experiments, no 2^nd^ or 3^rd^ jumps in the LSPRi response were observed, indicating a single binding event. Many nanosensors within the same array did not show any measurable response, serving as a negative-controls for the same experiment.

**Fig 6:**
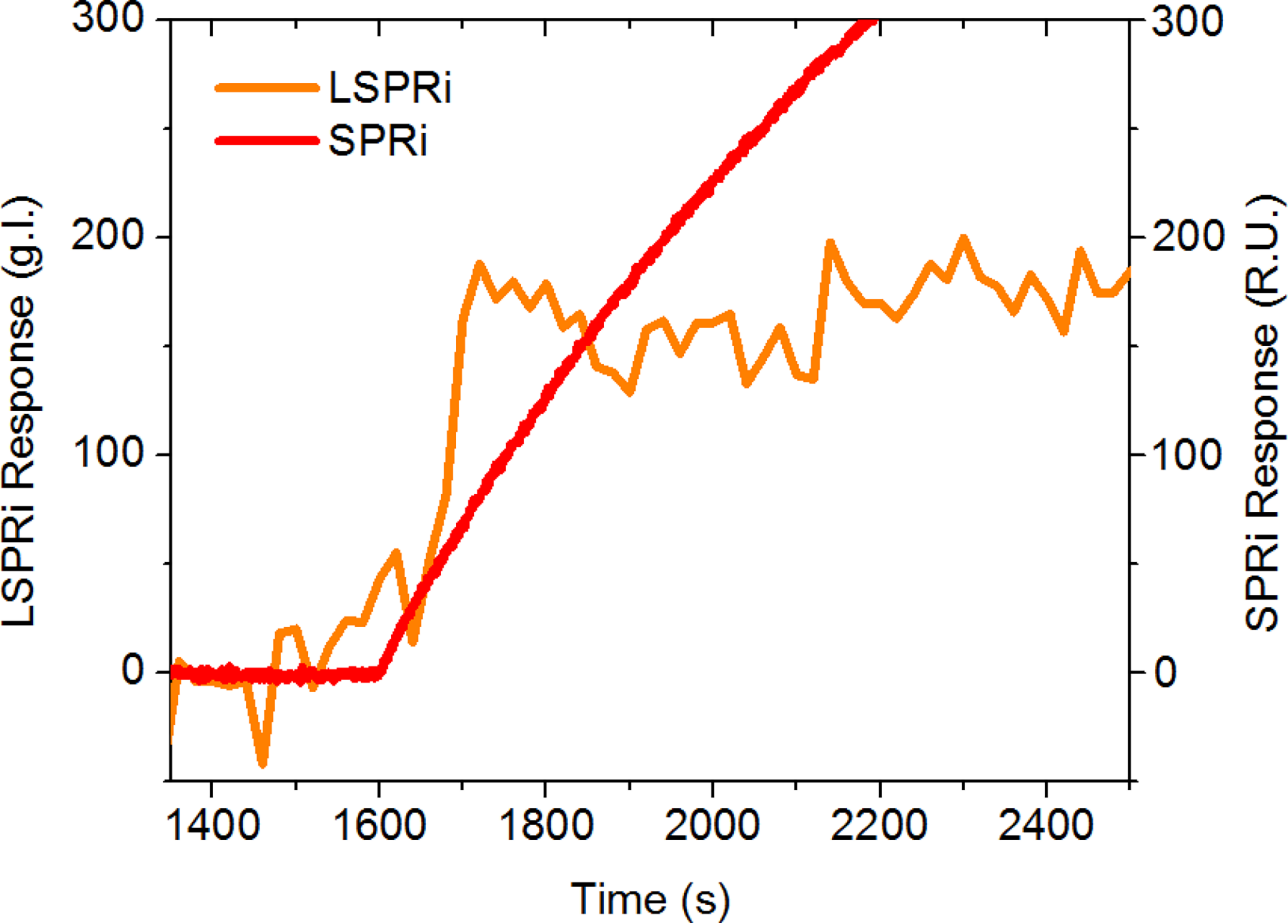
LSPRi versus SPRi exosome detection (background subtracted). LSPRi data (orange line) are from the green 4 × 4 subarray (5.8 μm^2^) of Fig 4 while the SPRi data (red line) are from a single sensor of the Biorad XPR36 SPRi system (250,000 μm^2^). In both experiments, an exosome concentration of 1 × 10^5^ exosomes/ml was introduced. The data sets have been offset in time so that the introductions of analyte coincide.

**Fig 6** compares the 16 nanopillar response from **Fig 4B** with that of a 500 μm × 500 μm Biacore SPRi sensor functionalized in the identical manner and subjected to the same exosome concentration. The SPRi sensor can accommodate millions of exosomes and as a result gives a smooth, integrated response. From this data no information can be gleaned regarding the size of the analyte. The nanopillars, in contrast, are designed for a digital response when exosome-sized particles bind to the surface, allowing us to visualize the stochastic nature of the sensing process, albeit with a noisier background. In addition, nanopillars which showed no response acted as size controls: if the analyte had been a protein, and therefore only a fraction of the sensor size, an integrated response would have been observed, much like that of the SPRi sensor [31]. The nanopillar design thus enables multiplexed biosensing, utilizing traditional control antibody approaches to test for specificity but also incorporates digital responses and size controls to improve confidence in exosome detection. The stochastic responses within the field of view were readily decoupled from global background contributions (*i*.*e*. focus drift) during the course of the experiment since all the nanostructures respond simultaneously to such global effects. The sensitivity of the technique presented here, along with its potential for multiplexing, will enable future exosome investigations in terms of characterization by cell type or by purification protocols which have been shown to alter exosome activity [32].

## 4. Conclusions

We have combined both nano- and microfabrication techniques to develop novel nanosensors tailored for exosome detection. By sizing the individual sensors to approximately the same diameter as the exosomes, we were able to observe digital responses occurring stochastically in time and space, consistent with single exosome detection. Placing the sensing element atop quartz pillars served to isolate nanosensor binding events from non-specific adhesion to the nearby substrate. The imaging approach to detection enabled highly multiplexed measurements, with thousands of nanopillars monitored in parallel and opens the possibility of future investigations in which exosome secretions from live cells can be monitored in real time.

## Supporting information

S1 Fig (TIF)

S2 Fig (TIF)

S3 Fig (TIF)

S1 File (DOCX)

S1 Table (TIF)

S2 Table (TIF)
