# Supplementary figures and images for: Nanoplasmonic pillars engineered for single exosome detection
Source: PLoS One. 2018 Aug 24;13(8):e0202773. doi: 10.1371/journal.pone.0202773 (PMC6108516; doi:10.1371/journal.pone.0202773)

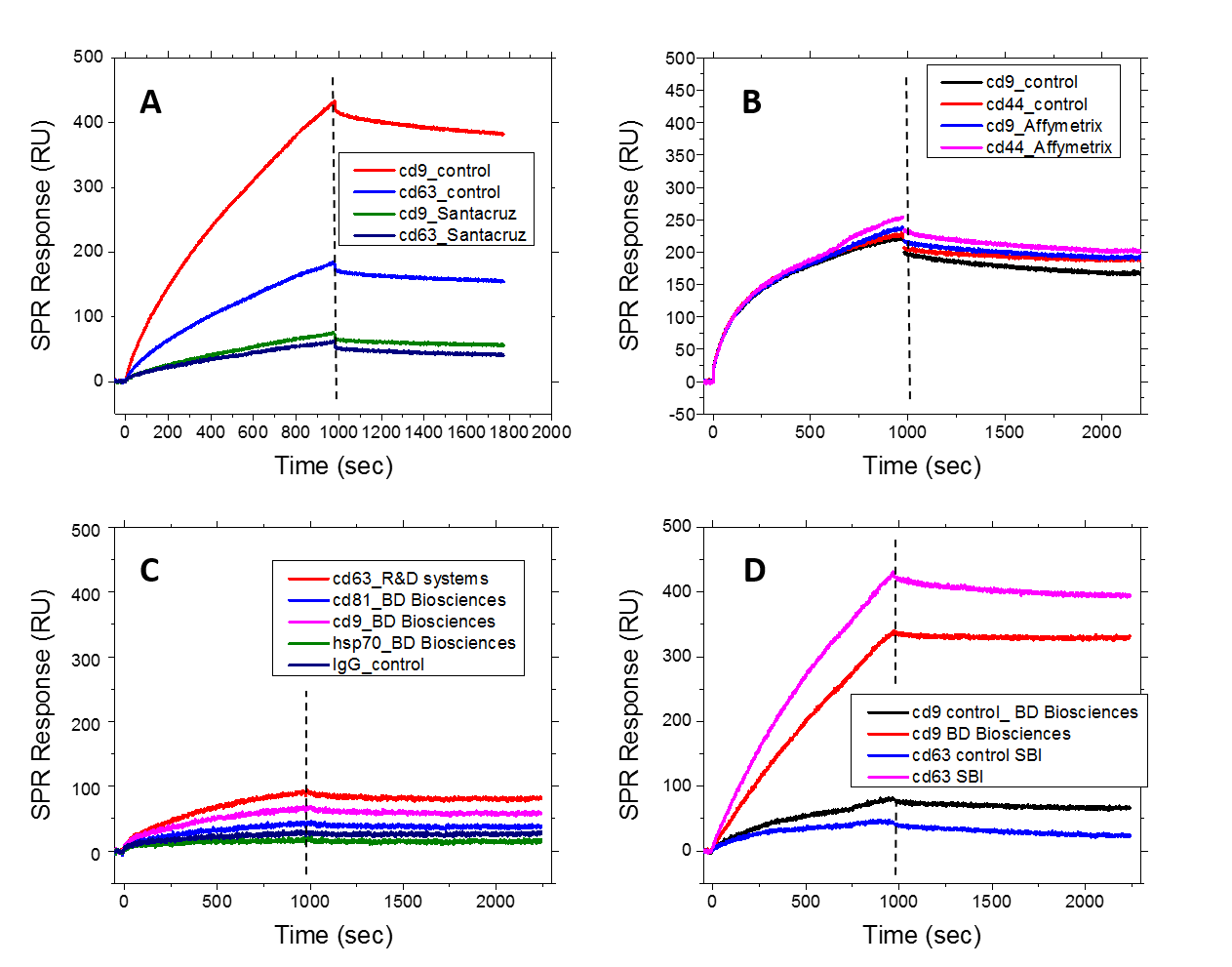

Supplement: S1 Fig — (TIF) [file pone.0202773.s001.tif]

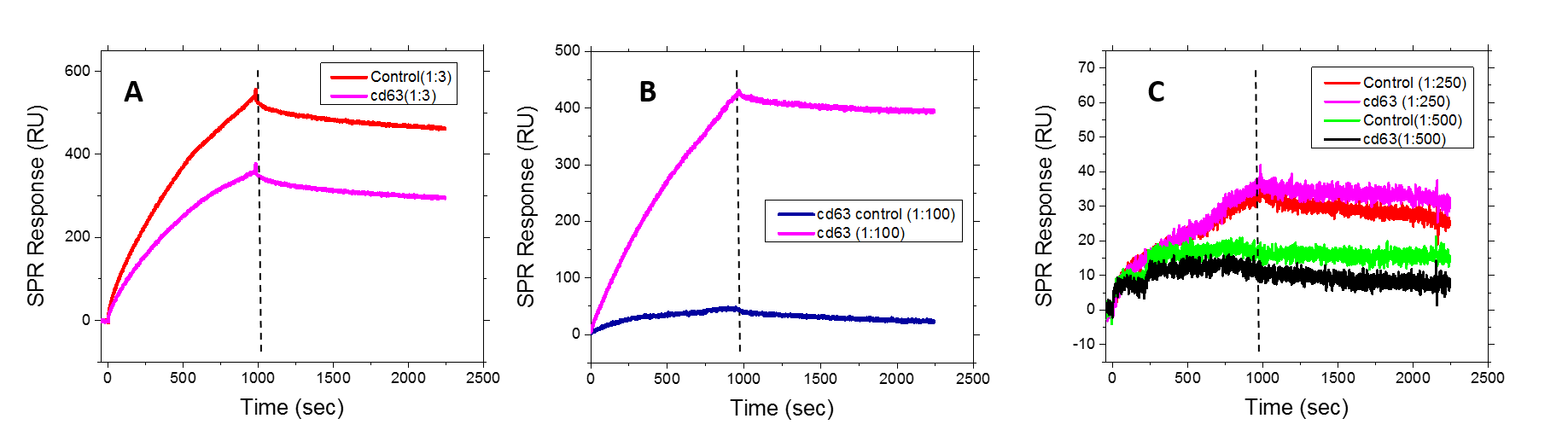

Supplement: S2 Fig — (TIF) [file pone.0202773.s002.tif]

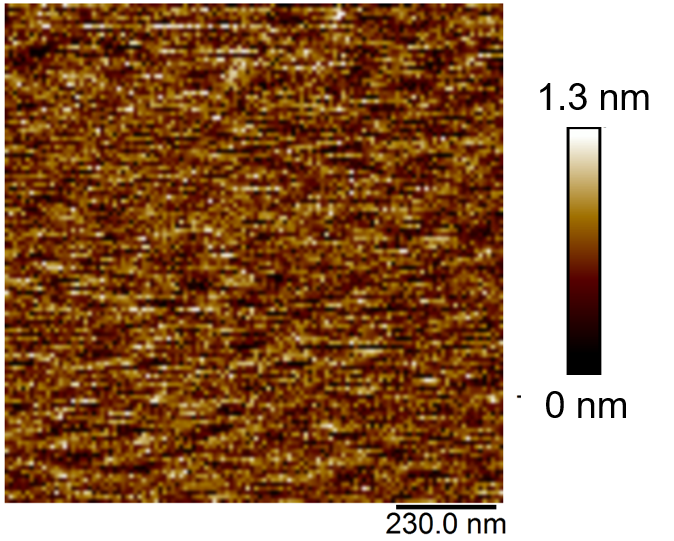

Supplement: S3 Fig — (TIF) [file pone.0202773.s003.tif]

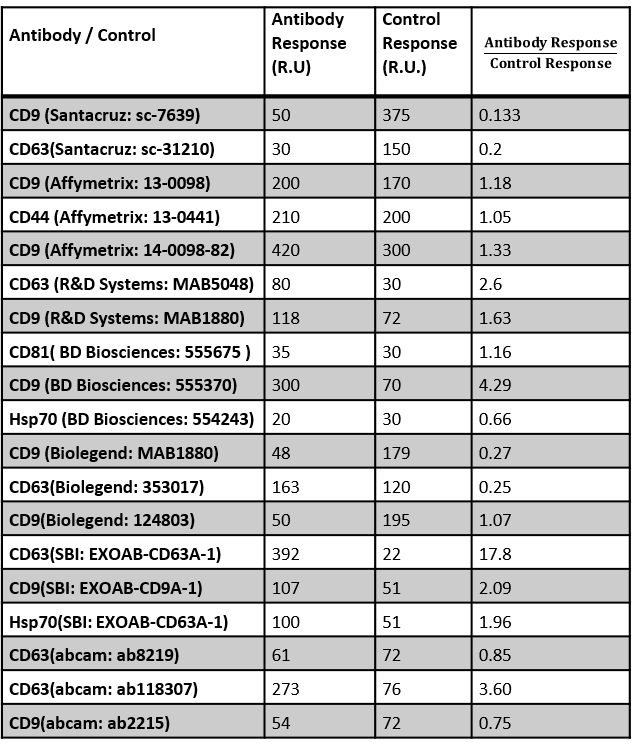

Supplement: S1 Table — (TIF) [file pone.0202773.s005.tif]

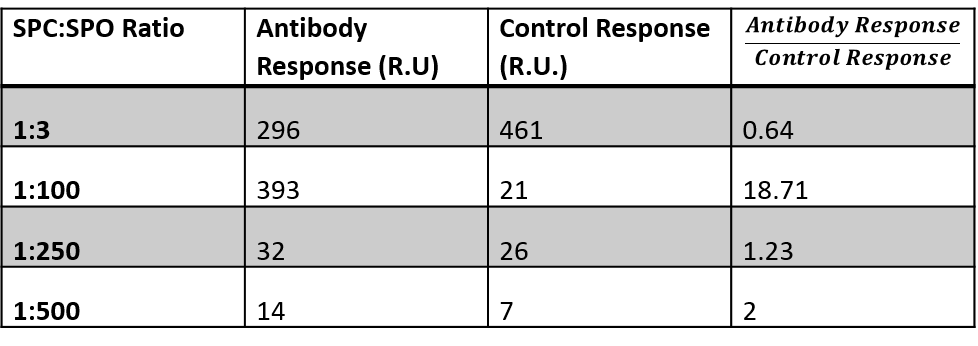

Supplement: S2 Table — (TIF) [file pone.0202773.s006.tif]
