# Supplementary material for: Nanoplasmonic pillars engineered for single exosome detection
Source: PLoS One. 2018 Aug 24;13(8):e0202773. doi: 10.1371/journal.pone.0202773 (PMC6108516; doi:10.1371/journal.pone.0202773)
Supplement: S1 File — (DOCX) [file pone.0202773.s004.docx]

**Supporting Information**

**SPR experiments**

To select a suitable antibody with the highest response to MCF7 exosomes, a total of 19 different antibodies from a variety of manufacturers were screened. All SPR experiments were conducted on a Biorad XPR36, and the sensor chips (ProteOn, Biorad) were cleaned by hydrogen plasma ashing and functionalized by the same protocol described in the main text. Supplementary Table 1 summarizes individual antibody’s response signal to MCF7 exosomes, their respective control response signal, and the ratio of the two.

Supplementary Figure 1 shows representative responses of MCF7 exosomes to various antibodies with their respective controls from different manufacturers. As shown in Supplementary Figure 1D, out of 19 different antibodies CD63 (SBI) and CD9 (BD Biosciences) had the highest specific response combined with the lowest non-specific binding. Based on this study, anti-CD63 (SBI) and associated rabbit IgG antibody control (targeted to Staphylococcal enterotoxin B) were chosen for all LSPRi exosome detection experiments.

SPR experiments were also performed to determine the SPC:SPO thiol ratio which maximized specific binding and minimized the non-specific binding response signals for exosomes using anti-CD63 (SBI) antibody. The ratio of SPC and SPO thiols was varied from 1:3 to 1:500 as summarized in Supplementary Table 2. Supplementary Figure 2 shows the corresponding SPR sensograms. As shown in Supplementary Figure 2B the 1:100 ratio of SPC: SPO gave the highest signal to non-specific binding ratio.

**LSPRi Analysis**

To analyze the LSPRi data over the course of hours during an experiment, the x-y drift was corrected using an ImageJ plugin time alignment tool (StackReg). Subsampling of the aligned imagery was accomplished with Matlab by superimposing a square grid on the nanosensors, the pixels’ intensity values in the region of interest (ROI) were then averaged for each time point of the two-hour experiment. The grid size is adjustable so that the number of nanopillars analyzed could vary from single nanosensor to the entire 10x10 or 20x20 nanosensor array. For example, Figure 3 in the main text shows grids sized to individual nanosensors, whereas Figure 4 shows grids sized to the entire nanosensors array as well subset ROIs of 4 x 4 nanosensor clusters.

**AFM**

To ensure AFM topographical data reflects the morphology rather than scan artifacts, a control experiment of freshly cleaved mica under 300uL of PBS is shown in Figure S3, exhibiting extremely flat surface with no particulates or aggregates.

**S1 Table**: MCF7 exosome response to various antibodies from different manufacturers and their respective control responses. CD9 (BD Biosciences) and CD63 (SBI), highlighted in red, exhibited the highest ratio of specific to non-specific binding response.

**S1 Fig**: (A)-(C): SPR sensograms showing binding of MCF7 exosomes to various antibodies from different manufacturers, and their respective control antibodies. The gray dashed line separates the association and dissociation phases. (D) Binding of exosomes to anti CD9 (BD Biosciences), anti CD63 (SBI), and their respective control antibodies that were chosen for this study.

**S2 Table:** Exosome response to anti-CD63 (SBI) and the control antibody for different SPC:SPO thiol ratios. SPC:SPO of 1:100 showed the highest ratio of specific to non-specific binding response (red).

**S2 Fig:** SPR sensograms showing the response of exosomes to different ratios of SPC to SPO thiols. (a) Response of exosomes to anti cd63 and control antibody on a surface functionalized with 1:3 ratio of SPC:SPO. (b) Response of exosome to anti-CD63 and control antibody on a surface functionalized with 1:100 ratio of SPC:SPO. (c) Response of exosome to anti cd63 and control antibody on a surface functionalized with 1:250 and 1:500 ratio of SPC:SPO. The gray dashed line separates the association and dissociation phases. 1:100 ratio shows the highest ratio of response for the specific to non-specific binding of exosomes

**S3 Fig:** Atomic Force Microscopy control scan of filtered PBS on mica.
